# Lipidomic Profiling of Liver Tissue from Obesity-Prone and Obesity-Resistant Mice Fed a High Fat Diet

**DOI:** 10.1038/srep16984

**Published:** 2015-11-23

**Authors:** Miso Nam, Myung-Sook Choi, Sunhee Jung, Youngae Jung, Ji-Young Choi, Do Hyun Ryu, Geum-Sook Hwang

**Affiliations:** 1 Integrated Metabolomics Research Group, Western Seoul Center, Korea Basic Science Institute, Seoul 120-140, Republic of Korea; 2 Department of Chemistry, Sungkyunkwan University, Suwon 440-746, Republic of Korea; 3 Department of Food Science and Nutrition, Kyungpook National University, Daegu, Republic of Korea; 4 Department of Life Science, Ewha Womans University, Seoul 120-750, Republic of Korea

## Abstract

Obesity is a multifactorial health problem resulting from genetic, environmental, and behavioral factors. A particularly interesting aspect of obesity is the differences observed in response to the same high-fat diet (HFD). In this study, we performed lipidomic profiling on livers from HFD-fed C57BL/6J mice using ultra-performance liquid chromatography–quadrupole time-of-flight mass spectrometry. Mice were divided into three groups: normal diet (ND), HFD-obesity prone (HFD-OP), and HFD-obesity resistant (HFD-OR). Principal components analyses showed a difference between the HFD-OP and HFD-OR groups. Individuals in the HFD-OR group were closer to those in the ND group compared with those in the HFD-OP group. In particular, phosphocholine (PC) and triglyceride (TG) levels differed significantly depending on the length of the acyl chain and degree of unsaturation, respectively. PC species were either positively or negatively correlated with concentrations of glucose, insulin, leptin, and hepatic cholesterol according to the length of the acyl chain. Decreased expression of the scavenger receptor B1 and ATP-binding cassette A1 in HFD-OP mice indicated that the acyl chain length of PC species may be related to high-density lipoprotein cholesterol metabolism. This study demonstrates that lipidomic profiling is an effective approach to analyzing global lipid alterations as they pertain to obesity.

Obesity rates have increased dramatically in recent years to become a serious health problem. Obesity is known to contribute to the development of nonalcoholic fatty liver disease (NAFLD), type 2 diabetes mellitus (T2DM), osteoarthritis, cardiovascular disease (CVD), and cancer^1^,^2^,^3^,^4^,^5^. Environmental influences like the excessive intake of fat and decreases in physical exercise may be key factors leading to obesity.

However, not all individuals become obese even when consuming a high-fat diet (HFD). Certain humans become obese (obesity-prone, OP), whereas others do not (obesity-resistant, OR), even when provided with the same HFD, indicating a difference in the metabolic response to HFD. Recent studies have suggested that a large number of factors, including differential regulation of intestinal lipid metabolism-related genes, changes in the expression levels of liver proteins, and metabolic adaptation, are important contributors to phenotypic development^6^,^7^,^8^. However, no lipid profiling studies have clearly demonstrated differences in lipid levels or lipid speciation. Lipid profiling of the aforementioned phenotypes would contribute greatly to our understanding of the physiological and biochemical differences between OP and OR individuals.

Lipid research is critical, given the extensive biological roles of lipids as signaling molecules, energy reserves, and structural components of biological membranes^9^. Lipidomics as a field aims to characterize lipid species and investigate the metabolic pathways and networks of lipids in biological systems. Lipidomics is used to discover and identify the structures of various lipid species, examine the distribution of lipids in biofluids and tissues, and study differences between lipids in different subject groups^10^,^11^,^12^.

Recent advances in analytical techniques have rejuvenated the field of lipid research at the molecular level. Global lipidomics allows for the rapid identification and relative quantification of lipid molecules across structural classes^12^,^13^ by employing mass spectrometry coupled to liquid chromatography (LC-MS)^14^,^15^ or shotgun analyses^16^,^17^. Although shotgun lipidomics is rapid and experimentally simple, it suffers from the risk of ion suppression and limitations in resolving isobaric compounds. In contrast, ion suppression and isobaric lipids are less of an issue for lipidomics using LC-MS. In particular, ultra-performance liquid chromatography–quadrupole time-of-flight MS (UPLC/QTOF MS), boasts high separation performance, increased sensitivity, high resolution, and the ability to characterize complex lipid species^18^,^19^.

Lipid profiling of HFD-induced obesity in C57BL/6J mice was performed to reveal differences in the levels of specific molecular lipid species in the livers of HFD-induced OP (HFD-OP) and OR (HFD-OR) phenotypes using UPLC/QTOF MS analyses of liver tissues.

## Results

### Animals and biochemical characteristics

**Figure 1:**
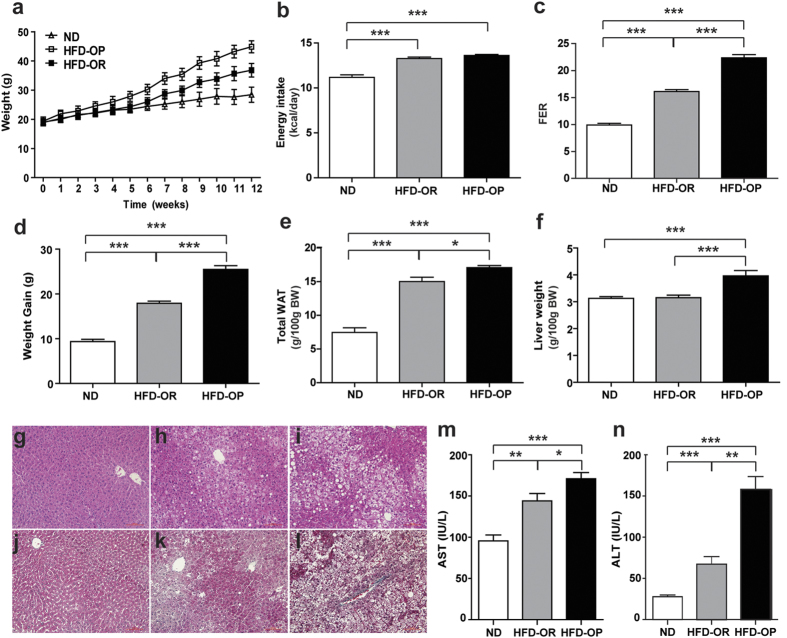
Changes in ND and HFD mice after 12 weeks. (**a**) Changes over 12 weeks in the body weights of ND, HFD-OP, and HFD-OR mice whose phenotype was observed after consuming a high-fat diet. Additional changes between ND and HFD mice after 12 weeks are shown for (**b**) average energy intake, (**c**) feed-efficiency ratio, (**d**) weight gain, (**e**) total white adipose tissue, and (**f**) liver weight. Representative H&E-stained liver tissues are shown for (**g**) ND, (**h**) HFD-OR, and (**i**) HFD-OP, indicating steatosis and hepatocyte ballooning. Masson’s trichrome stained liver tissue is also shown for (**j**) ND, (**k**) HFD-OR, and (**l**) HFD-OP, indicating hepatic fibrosis. Serum (**m**) AST and (**n**) ALT levels in ND and HFD mice are also shown. Data represent the mean ± SEM. Significant differences among groups were determined by Mann–Whitney *U*-tests. (*p < 0.05, **p < 0.01, ***p < 0.001).

Mice used in this study were randomly divided into two groups, with 10 mice fed a normal diet (ND) and 20 mice fed a high-fat diet (HFD). The HFD-fed mice were subdivided into HFD obesity-prone (HFD-OP) (n = 8) or HFD obesity-resistant (HFD-OR) (n = 8) groups, based on their body weight gain. The changes in body weight among ND, HFD-OP, and HFD-OR mice during the feeding duration are shown in Fig. 1a. At the beginning, all groups had comparable body weights. After 2 weeks of feeding, the HFD-OP mice were significantly heavier than mice in the other groups and increased in weight consistently until 12 weeks. The HFD-OR mice were significantly heavier than the ND mice after 6 weeks and continued to gain weight until 12 weeks. The average energy intake did not differ between HFD mice (Fig. 1b), whereas the feed-efficiency ratio of HFD-OP mice was significantly higher than that of HFD-OR mice (p < 0.001; Fig. 1c). Body weight gain was highest in the HFD-OP mice, followed by HFD-OR and ND mice (Fig. 1d). Body weight gain in HFD-OP mice was significantly higher than that in HFD-OR mice (p < 0.001). The total white adipose tissue weight per body weight for HFD-OP mice was significantly higher than that for HFD-OR mice (p < 0.05; Fig. 1e). Liver weight was also highest in the HFD-OP mice and differed significantly between the HFD-OP and HFD-OR mice (p < 0.01; Fig. 1f).

Liver lipid deposition and hepatic steatosis was monitored in each group using hematoxylin and eosin (H&E) staining (Fig. 1g-i). ND mice had no lipid deposition in the liver and exhibited normal tissue morphologies (Fig. 1g). Conversely, HFD mice had higher proportions of lipid droplets and developed steatosis and hepatocyte ballooning in the liver. Lipid deposition and inflammatory cell infiltrates and hepatocyte degeneration was more extensive in the liver of HFD-OP mice compared with HFD-OR mice (Fig. 1h,i). The development of hepatic fibrosis was confirmed by Masson’s trichrome staining (Fig. 1j–l). Collagen deposition and the development of hepatic fibrosis in necrotic areas of liver tissues were also higher in HFD-OP mice compared with HFD-OR mice (Fig. 1k,l). Among the HFD mice, the plasma aspartate aminotransferase (AST) and alanine aminotransferase (ALT) levels of HFD-OP and HFD-OR mice differed significantly (p < 0.05, p < 0.01 respectively; Fig. 1m,n).

The biochemical characteristics of ND, HFD-OP, and HFD-OR mice are shown in Table 1. In plasma, the concentrations of total cholesterol (total-C), HDL cholesterol (HDL-C), and non-HDL-cholesterol (non-HDL-C) were higher in HFD-OP mice than in HFD-OR mice. The levels of apolipoprotein A1 (apo-A1) in the HFD-OP mice were similar to those in the HFD-OR mice. The levels of glucose, insulin, and leptin and the homeostasis model assessment for insulin resistance (HOMA-IR) were also significantly elevated in the HFD-OP mice. The concentration of triglycerides (TGs) was highest in HFD-OR mice plasma, followed by ND and HFD-OP mice. In liver tissues, hepatic TGs and hepatic cholesterol levels were significantly higher in HFD-OP mice than in HFD-OR mice (Table 1).

To investigate hepatic insulin resistance, we analyzed Akt phosphorylation in liver. The results showed that Akt phosphorylation was inhibited in HFD-OP mice (Supplementary Fig. S1).

### Lipid profiling by UPLC/QTOF MS

**Figure 2:**
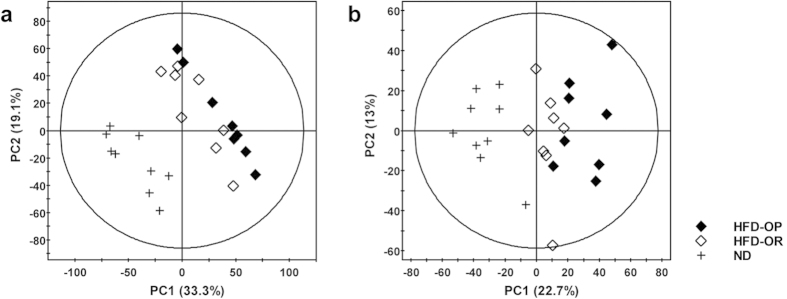
Multivariate statistical analyses of liver lipid extracts in ND and HFD mice. Principal component analysis (PCA) scatterplots obtained from the UPLC/QTOF MS spectra of liver lipid extracts for global analysis are shown for (**a**) positive and (**b**) negative modes. (**a**) Positive mode (*R*^2^*X* = 68.3%, *Q*^2^ = 49.5%), (**b**) negative mode (*R*^2^*X* = 60.3%, *Q*^2^ = 26.0%). *R*^2^*X* represents the goodness of fit and *Q*^2^ indicates the predictability of the models.

UPLC/QTOF MS data were used in principal component analysis (PCA) and partial-least-squares discriminant analysis (PLS-DA) analyses to compare the global lipid profiles of liver tissues from ND, HFD-OP, and HFD-OR mice after 12 weeks. The PCA (Fig. 2a,b) and PLS-DA (Supplementary Fig. S2a,b) score plots show a significant separation among the three groups. The PCA models were established using five components in both positive (R2X = 68.3%, Q2 = 49.5%) and negative modes (R2X = 60.3%, Q2 = 26.0%). In both modes, the ND mice were separated from the HFD mice by the first component. The PCA score plot also shows that the HFD-OR mice were closer to the ND mice than to the HFD-OP mice. PLS-DA models were used to maximize the lipid metabolite pattern among the groups. The PLS-DA score plots showed a clear separation between the HFD-OP and HFD-OR groups in both positive and negative mode (Supplementary Fig. S3a,b).

A VIP score of >1 in the PLS-DA model and independent t-tests were used as selection criteria to determine the specific lipid species associated with the differentiation of HFD-OP and HFD-OR mice liver tissues. As shown in Supplementary Table S2, 59 features were finally identified, including the sphingolipids such as ceramide (Cer) and sphingomyelin (SM); glycerophospholipids including lysophosphatidylcholine (lysoPC), lysophosphatidylethanolamine (lysoPE), PC, PE, phosphatidylinositol (PI), and phosphatidylserine (PS); and glycerolipids including diglyceride (DG) and TG.

**Figure 3:**
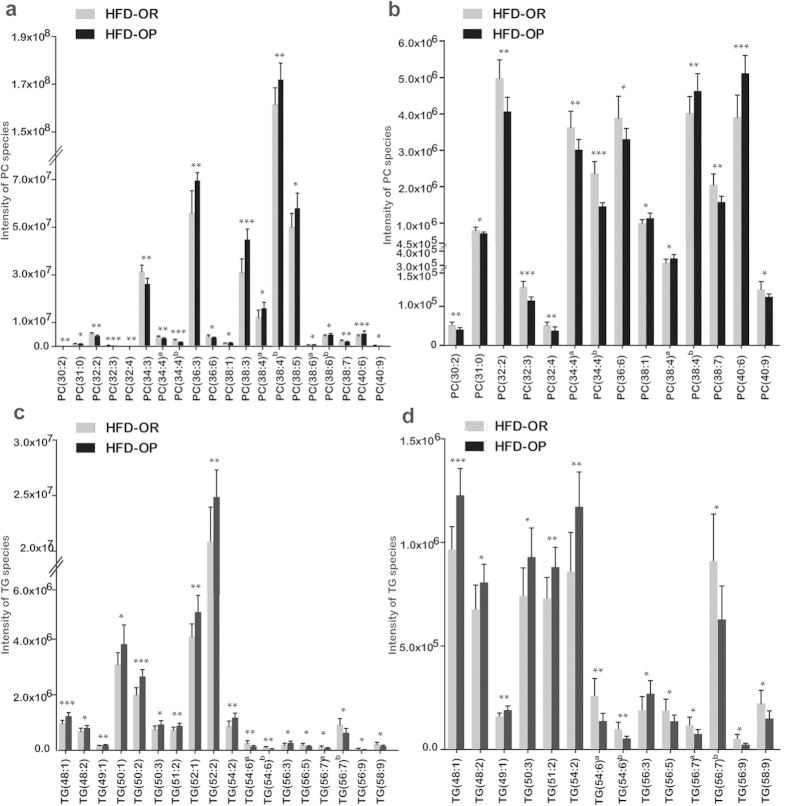
Intensities of PC and TG species from HFD-OR and HFD-OP mice. (**a**) The intensities of all PC species in liver tissue, and (**b**) the intensity of each PC species not verified in (**a**). (**c**) The intensity of all identified TG species in liver tissue, and (**d**) the intensity of each TG species not verified in (**c**). The presented PC and TG species are showed significant differences between the HFD-OP and HFD-OR groups which were determined by Mann-Whitney U-tests. (*p < 0.05, **p < 0.01, ***p < 0.001).

A heat map used to evaluate lipid patterns (Supplementary Fig. S4) showed that the levels of lipid metabolites in the livers of HFD-OR mice were similar to those of ND mice. PC and TG levels in liver were higher or lower in the HFD-OP and HFD-OR mice depending on the acyl chain length and the degree of unsaturation, respectively (Fig. 3). The levels of PC species with relatively long acyl chains (>36 carbon atoms) were relatively low, and the levels of PC species with short acyl chains (<34 carbon atoms) were relatively high in HFD-OR mice. Levels of PC species with high degrees of unsaturation, such as PC 40:9 and PC 38:7, were high in HFD-OR mice despite long acyl chain lengths. The levels of TG species having low degrees of unsaturation (<4) were high in the HFD-OP groups compared with the HFD-OR mice. Conversely, HFD-OR mice had higher levels of TG species with high degrees of unsaturation (>4).

### Correlations of PC species with biochemical parameters

**Figure 4:**
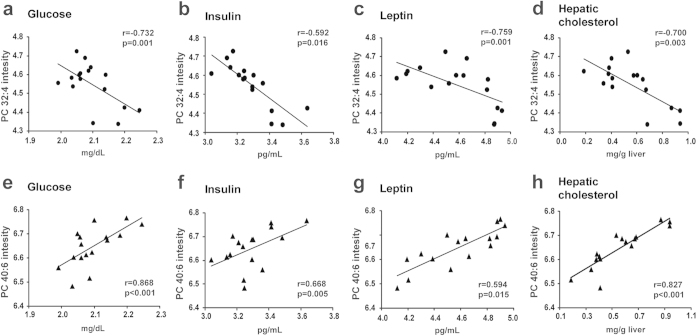
Correlation of PC species and biochemical parameters. Representative Spearman’s rank correlations are shown for PC 32:4 and PC 40:6 with different biochemical characteristics: (**a,e**) glucose, (**b,f**) insulin, (**c,g**) leptin, and (**d,h**) hepatic cholesterol.

Correlations between identified PC species and the biochemical parameters of plasma and liver tissues were determined in both HFD-OP and HFD-OR mice. Representative Spearman’s rank correlations for PC 32:4 and PC 40:6 with different biochemical characteristics are shown in Fig. 4. Glucose concentrations were negatively correlated with short acyl chain lengths, and PC 32:4 was lower in HFD-OP mice than in HFD-OR mice (Fig. 4a). Conversely, glucose concentrations were positively correlated with long acyl chain lengths and was higher for PC 40:6 in HFD-OP mice than in HFD-OR mice (Fig. 4e). The levels of insulin, leptin, and hepatic cholesterol were correlated in the same way as glucose (Fig 4b–d,f–h). Supplementary Fig. S5 shows the positive correlation between long PC acyl chain lengths and glucose, insulin, leptin, and hepatic cholesterol.

### Decreased SR-B1 and ABCA1 hepatic protein levels and mRNA in HFD-OP mice

**Figure 5:**
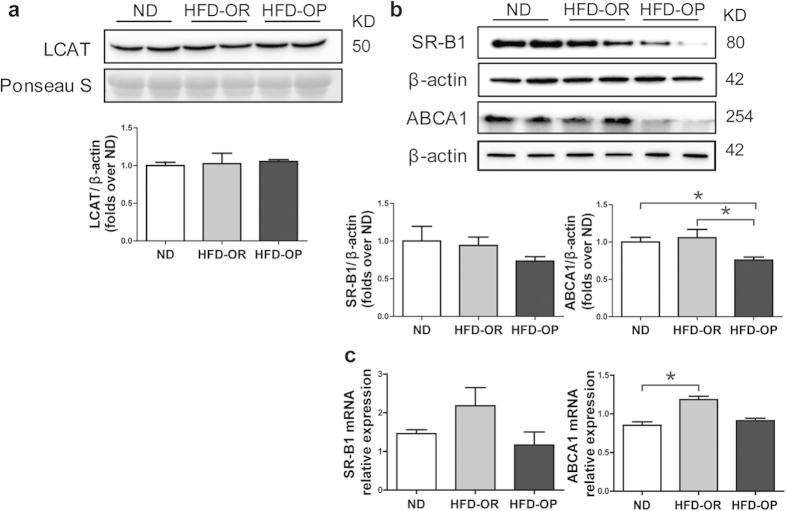
Expression of HDL-related genes in ND and HFD mice. (**a**) Representative western blot analysis and quantification of the expression of LCAT in mouse plasma (ND, n = 5; HFD-OP, n = 5; HFD-OR, n = 5). Ponceau S staining serves as the loading control. (**b**) Representative western blot analysis and quantification of expression of SR-B1 and ABCA1 (ND, n = 5; HFD-OP, n = 5; HFD-OR, n = 5). β-Actin was used as a loading control. (**c**) Transcript levels of SR-B1 and ABCA1 (ND, n = 5; HFD-OP, n = 5; HFD-OR, n = 5). mRNA levels were normalized to those of GAPDH. Data represent the mean ± SEM. Significant differences among groups were determined by Mann–Whitney *U*-tests. (*p < 0.05).

Previous studies have suggested that the HDL phospholipid acyl chain composition affects the efflux of cellular free cholesterol^20^. Our results show that the levels of hepatic cholesterol correlated positively or negatively with PC levels, depending on whether the acyl chain of the PC species was long or short, respectively. We examined the effect of liver PC acyl chain length on the expression of lecithin-cholesterol acyltransferase (LCAT), scavenger receptor B1 (SR-B1), and ATP-binding cassette transporter A1 (ABCA1), which are related to HDL-C metabolism. Western blot analyses showed that the expression of LCAT in plasma was unchanged (Fig. 5a), whereas the expression levels of both SR-B1 and ABCA1 were markedly lower in the livers of HFD-OP mice compared with those of HFD-OR mice (Fig. 5b). The mRNA levels of ABCA1 and SR-B1 also trended lower in HFD-OP mice compared with HFD-OR mice; however, the differences were not statistically significant. These results indicate that differences exist in the capacity of cholesterol efflux between these two groups. In addition, the cholesterol biosynthetic pathway through quantitative PCR of SREBF1 and SREBF2 was examined, but there was no difference between HFD-OP and HFD-OR mice (data not shown).

## Discussion

In biological systems, lipids comprise cell membranes, determine membrane dynamics, help maintain energy homeostasis, and play important roles in cell signaling^9^. Thus, abnormal lipid metabolism is associated with diverse disease states. In the present study, we applied global lipid profiling to liver tissues to further clarify the metabolic differences between HFD-OP and HFD-OR mice. The mice were divided into HFD-OP or HFD-OR groups based on body weight distribution of HFD mice. Weights of the liver and total white-adipose tissue and levels of glucose, insulin, leptin, and total cholesterol showed significant differences between the HFD groups. In PCA score plots derived from UPLC/QTOF MS spectra, the ND mice and HFD mice were clearly distinguished. HFD-OR mice were closer to the ND mice compared with the HFD-OP mice, indicating that the lipid composition of HFD-OR mice liver was more similar to that of ND mice than that of HFD-OP mice. Levels of sphingolipids, glycerophospholipids, and glycerolipids differed significantly between HFD-OP and HFD-OR mice, and variations in their relative levels are associated with obesity.

The compositions and concentrations of lipids in rodent and human obesity models have been measured previously. Pietiläinen *et al.* profiled lipids in monozygotic twins and showed upregulated levels of lysoPC and downregulated levels of ether phospholipids in the sera of obese twins^21^. Barber *et al.* reported that levels of lysoPC species in mouse plasma decreased, and that levels of SM and Cer increased after 1 week of being fed a HFD^22^. Several reports, however, provide conflicting results with regard to the lipid composition of liver tissues in specimens fed an HFD. Lipid profiling of liver tissues has shown elevated levels of TG and Cer species^14^. In contrast, Eisinger *et al.*^17^ reported normal SM levels, whereas monounsaturated Cer and hexosylceramide levels decreased along with lower concentrations of PC, PE, and PS species. Thus, non-uniform changes in lipid composition occurring in various obesity models is not surprising. We also monitored changes in the levels of several lipid classes in HFD-OP and HFD-OR mice. The concentrations of some lipid species agreed with previous results, whereas others did not. Note that elucidating changes in lipid species is complicated by the many types of obesity models. Furthermore, only one lipid metabolite of each lipid class was identified, such as PI 38:3, PS 38:6, or SM d34:2, and may not be representative of the entire class.

The total TG levels in both liver tissue and plasma generally increased in rodent and human obesity models^21^,^23^,^24^. However, the relative TG composition varied with the obesity model. In a diet-induced weight loss model, weight reduction in mice was associated with lower levels of TG containing saturated fatty acids, e.g., TG species with acyl chains 16:0/14:0/14:1, 16:0/16:0/16:0, 18:1/14:0/16:0, 16:0/18:0/16:0, 16:0/16:0/18:1, 18:1/16:0/16:1, and 16:0/18:1/18:0 relative to a control group^25^. Levels of serum TG species in individuals with nonalcoholic fatty-liver disease were positively associated with saturated or monounsaturated fatty acid TGs and negatively associated with polyunsaturated fatty acid-containing lipids such as PC, PE, and TG species^26^. Levels of TG 52:2 and TG 54:3, which contain relatively short-chain fatty acids, were twofold higher in the ob/ob adipose tissue of HFD mice relative to wild-type mice. Also, the level of TG 54:6, which is composed of polyunsaturated fatty acids, decreased by 40% in ob/ob mice^27^. We found that the levels of TG species with less than four degrees of unsaturation were higher in HFD-OP mice than in HFD-OR mice, and that levels of TG species with greater than four degrees of unsaturation were higher in HFD-OR mice, showing that differences in the degree of saturation of TG species in the liver affect the individual response to HFD.

In the above results, the dependencies of PC levels on acyl chain length differed between the HFD-OP and HFD-OR mice. The acyl chain length of PC species can be correlated with differences in several classical plasma biochemical parameters. Acyl chain length correlated either positively or negatively with concentrations of glucose, insulin, and leptin, high levels of which were observed in HFD-OP mice. Matsuzaka *et al.*^28^ suggested that Elvovl6, a gene involved in the formation of long-chain fatty acids by encoding elongase, plays a role in the elongation of palmitate (16:0) to stearate (18:0). Mice deficient in *Elvovl6* showed lesser frequencies of hyperglycemia, hyperinsulinemia, and hyperleptinemia and could regulate insulin sensitivity through adjustments in their hepatic fatty acid compositions. In addition, phospholipid chain length has an effect on pulmonary insulin absorption^29^. Therefore, PC acyl chain length can affect concentrations of glucose, insulin, and leptin. Increased proportions of short fatty acid PC species, such as those found in HFD-OR mice, may improve insulin and leptin regulation and enhance obesity resistance.

The levels of insulin and HOMA-IR were the same in HFD-OR and ND mice, whereas they were higher in HFD-OP mice, suggesting that HFD-OP mice possessed insulin resistance. Inhibition of Akt phosphorylation also supported insulin resistance in HFD-OP mice. Although HFD-OP and HFD-OR mice consumed the same diet, only HFD-OP mice showed insulin resistance and hyperinsulinemia-mediated increases in hepatic *de novo* lipogenesis (DNL). In states of insulin resistance, elevated insulin levels do not fully suppress the hormone-sensitive lipase (HSL), which hydrolyzes free fatty acids from adipocyte TG and activates lipoprotein lipase (LPL)^30^. As a result, an influx of fatty acids to the liver increases *de novo* lipogenesis (DNL). The accumulation of hepatic TG and the histology of liver tissues in HFD-OP mice also suggest the increase in DNL. Activation of HSL and LPL may result in lower levels of circulating TG in HFD-OP mice compared with HFD-OR mice by clearing TG-rich plasma lipoproteins and hydrolyzing the TG in adipocytes.

The present study also indicates that PC acyl chain length in the liver is associated with levels of hepatic cholesterol. HFD-OP mice showed higher levels of hepatic cholesterol and plasma total-C, which indicates that cholesterol metabolism was abnormal in the HFD-OP mice. In a previous study, the HDL phospholipid fatty acyl chain composition was shown to affect the efflux of cellular free cholesterol^20^. Therefore, one can reasonably assume that PC acyl chain length may influence cholesterol efflux.

HDL-C acts as a reverse cholesterol transport (RCT), the anti-atherogenic mechanism by which excess free cholesterol in peripheral tissues is transported to the liver for removal and recycling^31^. Despite higher levels of hepatic cholesterol and total-C, no changes in the plasma levels of apoA-1 and HDL-C were observed in the HFD-OP mice. The expression level of LCAT, which plays a central role in maintaining HDL-C levels in the plasma, was likewise unchanged, suggesting that free cholesterol was converted to cholesteryl esters (CE) by the enzyme LCAT and led to HDL maturation within the HDL particle.

The expression of both SR-B1 and ABCA1 was low in the livers of HFD-OP mice. SR-B1 is an integral membrane protein expressed abundantly in several tissues, including the liver, and is a major HDL-C receptor mediating the selective uptake of CE^32^. ABCA1 is a transporter central to the synthesis of HDL by mediating the efflux of cholesterol and phospholipids to lipoproteins. Hepatic ABCA1, the most important modulation factor of hepatic cholesterol efflux, induces pathways that regulate cholesterol homeostasis in the liver^33^. *In vivo* modulation of HDL phospholipid content affects SR-B1- and ABCA1-mediated cholesterol efflux^34^. HDL phospholipids composed of long acyl chains showed lower capacities for the efflux of cellular free cholesterol^20^. Thus, the observed high proportions of long acyl chain PCs in HFD-OP mice liver may affect the levels of HDL phospholipids, causing abnormal HDL-C metabolism. Vergeer *et al.* reported that a mutation of SR-B1 in humans caused increased HDL-C levels and a reduced capacity for cholesterol efflux from macrophages without increasing the severity of atherosclerosis^35^, which may explain why concentrations of HDL-C were higher in HFD-OP mice, whereas the HDL-C/total-C ratio (HTR) was higher in HFD-OR mice. Moreover, SR-B1 knockout mice showed increased levels of plasma cholesterol^36^. Note that SR-B1 protects against atherosclerosis^37^. The significant lipid deposition was observed in various tissues of ABCA1-KO mice^38^. In addition, overexpression of human ABCA1 in C57BL/6 mice resulted in anti-atherogenic profile with decreased plasma cholesterol, cholesteryl ester, free cholesterol, and non HDL-C levels, but with increased HDL-C, apoA-I, and apoE levels^39^. Therefore, elevated ABCA1 expression in HFD-OR mice demonstrates that HFD-OR mice are more anti-atherogenic compared to HFD-OP mice, and it causes low levels of total cholesterol and non HDL-C and high level of HTR in HFD-OR mice. Our results therefore indicate that HFD-OP mice may be more vulnerable to atherosclerosis than HFD-OR mice.

SR-B1 and ABCA1 are also integral membrane proteins, and PC species are the primary components of cell membranes. Therefore, the PC composition of liver tissues may affect the expression of SR-B1 and ABCA1 since it is so similar to the PC composition of hepatocyte membranes.

This study was performed in mice livers, which are not entirely consistent with human NAFLD or nonalcoholic steatohepatitis (NASH). Nevertheless, the results from several lipidomics studies of humans with NAFLD or NASH are similar to those presented here. Puri *et al.*^40^ showed that polyunsaturated fatty acid-containing lipids such as TG and PC were lower in NASH livers. Similarly, polyunsaturated fatty acid-containing PE and PC species were reduced in cirrhotic human liver samples, whereas saturated, mono- and di-unsaturated fatty acid-containing PE and PC species were increased^41^. Thus, this study adds to our understanding of NAFLD.

In conclusion, the liver tissues of HFD-OP and HFD-OR mice models were compared and characterized using lipid profiles to provide new insights into lipid metabolism. Significant differences in the relative concentration of lipid species were observed between HFD-OP and HFD-OR mice. This study showed that UPLC/QTOF MS-based lipid profiling is an effective analytical tool for the characterization of lipid composition in HFD-OP and HFD-OR mice.

## Methods

### Animals and experimental design

Four-week-old male C57BL/6J mice were obtained from Jackson Laboratories (Bar Harbor, ME, USA). All mice were housed individually under a constant temperature (24 °C) and on a 12-h light/dark cycle. Upon arrival, mice were fed a normal chow diet for 1 week to facilitate acclimation. At 5 weeks of age, mice were randomly divided into normal diet (ND, n = 10) and high-fat diet (HFD, n = 20) groups. The ND feed (TD94045, Harlan, Madison, WI, USA) consisted of 17.2 kcal% fat, 18.8 kcal% protein, and 63.9 kcal% carbohydrate. The HFD feed (TD06414) consisted of 60.3 kcal% fat, 18.4 kcal% protein, and 21.3 kcal% carbohydrate (Supplementary Table S1).

After 6 weeks on the HFD, identification of HFD-OP and HFD-OR mice was based on body weight gain. Mice were subdivided into HFD-OP (n = 8, >21 g) and HFD-OR (n = 8, <20 g) groups according to the mean value of 20.5 g. Four mice with a body weight gain between 20 and 21 g were excluded from the study as they prevented a clear distinction between the groups. All mice were fasted overnight in individual cages before being sacrificed. Blood and liver tissues were harvested from the sacrificed animals and stored at −80 °C. Body weight and food intake was measured twice per week for each mouse.

All studies were performed in accordance with the protocols for animal studies approved by the ethics committee of Kyungpook National University (KNU 2012-136).

### Analysis of plasma lipids

Enzymatic assays for plasma total-C, HDL-C, and TGs were performed using enzymatic kits (Asan Pharmaceutical Co., Seoul, Korea). Apo-A1 levels were also measured using enzymatic kits (Eiken, Tokyo, Japan). Non-HDL-C was calculated as (total-C) – (HDL-C).

### Fasting blood glucose and insulin sensitivity

The fasting blood glucose concentration was measured using the glucose analyzer, GlucDr Supersensor (Allmedicus, Korea) in whole blood obtained from the tail vein after 12-h fasting. Plasma insulin was quantified using a multiplex detection kit (171-F7001M, Bio-Rad, Hercules, CA, USA) according to the manufacturer’s instructions. HOMA-IR was calculated as follows: HOMA-IR = [fasting glucose (mmol/L) × fasting insulin (mU/mL)]/22.5.

### Plasma glucagon and leptin measurements

Plasma hormones (glucagon) and adipokines (leptin) were measured with a multiplex detection kit (Bio-Rad, Hercules, CA, USA). Capture antibodies directed against the glucagon and leptin were covalently coupled to beads, and the coupled beads were exposed to the test plasma. After a series of washes to remove unbound protein, a biotinylated detection antibody was added to create a sandwich complex. The final detection complex was formed with the addition of a streptavidin–phycoerythrin conjugate, with phycoerythrin serving as the fluorescent indicator. All samples were assayed in duplicate and analyzed with a Luminex 200 Labmap system (Luminex, Austin, TX, USA). Data analyses were performed with Bio-Plex Manager software version 4.1.1 (Bio-Rad).

### AST and ALT activities

AST and ALT activities were measured using a commercially available kit (Asan Pharm Co., Seoul, Republic of Korea) following the manufacturer’s instructions.

### Histological analysis of liver tissue

Liver tissue was excised from each mouse and subsequently fixed in 10% (v/v) paraformaldehyce/PBS. Samples were then embedded in paraffin for staining with H&E and Masson’s trichrome dye. The stained slices were examined under an optical microscope (Zeiss Axioscope) at 200x magnification^42^.

### Hepatic lipid profiles

Hepatic lipids were extracted^43^, and the dried lipid residues were dissolved in 1 mL of ethanol for TGs and cholesterol assays. Triton X-100 and a sodium cholate solution in distilled water were added to 200 *μ*L of the dissolved lipid solution for emulsification. The TGs and cholesterol concentrations were determined using the same enzymatic kit used for plasma analyses.

### Liver tissue extraction

Liver tissue (60 mg) was transferred to a 1.5-mL tube containing 2.8-mm zirconium oxide beads and homogenized twice using a Precellys 24 tissue grinder (Bertin Technologies, Ampère Montigny-le-Bretonneux, France) at 5000 rpm with 1.5 mL of a 1:1 v/v methanol:water mixture for 15 s. After homogenization, the mixture was centrifuged for 10 min at 16,000 rpm at 4 °C. The supernatant was then transferred into a 1.5-mL Eppendorf tube, and 1.6 mL of a 3:1 v/v chloroform:methanol mixture was added to the solid precipitate. The mixture was homogenized twice at 5000 rpm for 15 s and centrifuged for 10 min at 16,000 rpm at 4 °C. The supernatant was transferred to a 1.5-mL Eppendorf tube and evaporated under a stream of nitrogen. For the UPLC/QTOF MS analyses, liver lipid extracts were diluted with an isopropanol:acetonitrile:water mixture (2:1:1, v/v/v).

### Lipid profiling

Liquid chromatography-electrospray ionization–tandem MS (LC-ESI-MS/MS) analyses of liver lipid extracts were performed on a triple TOF™ 5600 MS/MS System (AB Sciex, Concord, ON, Canada), combined with a UPLC system (Waters, Milford, MA, USA). Separations were performed on an Acquity UPLC BEH C18 column (2.1 × 100 mm with 1.7-*μ*m particles; Waters). The column oven temperature was maintained at 40 °C, and the autosampler temperature was held at 4 °C. The injection volume of the sample was 3 *μ*L using a partial loop mode. The binary gradient system comprised 10 mM ammonium acetate in an acetonitrile:water mixture (40:60, v/v; solvent A) and 10 mM ammonium acetate in an acetonitrile:isopropanol mixture (10:90, v/v; solvent B). The gradient profile was 40–65% B over 5 min, 75% B at 20 min, 99% B at 25 min, 99% B from 25 to 27 min, and 40% B from 27.1 to 29 min to equilibrate the separation system for subsequent runs. The flow rate was kept at 0.35 mL/min for 29 min.

UPLC/QTOF MS spectral data were analyzed by MarkerView^TM^ (AB Sciex), which was used to find peaks, perform the alignment, and generate peak tables of *m/z* and retention times (min). Spectra were normalized to the total spectral area. To determine the precision of detection, lipid metabolites with coefficients of variation below 20 were selected.

Lipids were putatively identified by comparing the experimental data against various lipid metabolite databases, including the lipidmap (www.lipidmaps.org), metlin (metlin.scripps.edu), and human metabolome (www.hmdb.ca) databases. The isotope pattern matching and fragment patterns (MS/MS spectra) were similarly used to identify lipid metabolites. LysoPC^44^, PC and SM species^45^,^46^ showed distinct fragmentation patterns: an abundant product ion of *m/z* 184 in tandem mass spectra of positive ESI mode. Neutral loss fragments of m/z 141 were used for identification of lysoPE and PE^47^,^48^. PI, PS and Cer were analyzed by neutral loss fragments of 277, 185, and 264, respectively^46^,^49^.

### Statistical analysis

Statistical analyses were performed using the Mann–Whitney *U*-test with the Statistical Package for Social Sciences software, version 15.0 (SPSS Inc., Chicago, IL, USA). A two-tailed *p*-value of <0.05 was considered statistically significant. All results are presented as the average ± standard deviation (SD). Correlations between specific lipid metabolites and the concentrations of glucose, insulin, leptin, and hepatic cholesterol were evaluated using this same software. Multivariate statistical analyses were performed with a unit variance scale using SIMCA-P^+^ software, version 12.0 (Umetrics, Umeå, Sweden). Heat maps were generated using MultiExperiment Viewer Ver. 4.9.0 (Mev, http://www.tm4.org/mev/).

### Western blot analysis

The expression levels of Akt, phosphor-Akt (p-Akt), LCAT, SR-B1, and ABCA1 in mouse plasma (1 *μ*L) and liver extracts (50 *μ*g) were analyzed by Western blot analyses. Protein extracts were prepared in RIPA buffer with a protease inhibitor cocktail (BioVision, Mountain View, CA, USA). Proteins were harvested and centrifuged for 15 min at 4 °C. The supernatant was saved, and the protein concentration was determined with a BCA protein assay. Proteins were separated by sodium dodecyl sulfate–polyacrylamide gel electrophoresis (SDS-PAGE) and transferred to a polyvinylidene difluoride membrane (GE Healthcare, Milwaukee, WI, USA). The membranes were then washed with phosphate-buffered saline (PBS) supplemented with 0.05% (v/v) Tween 20 (PBS-T) followed by blocking with PBS-T containing 5% (w/v) nonfat dry milk. The membranes were incubated for 2 h at room temperature with antibodies specific for Akt (1:1000 dilution; Cell signaling, Beberly, MA, USA), p-Akt (1;500 dilution; Cell signaling), LCAT (1:500 dilution; Abcam, Cambridge, MA, USA), SRB-1 (1:1000 dilution; Abcam), and ABCA1 (1:500 dilution; Abcam). Membranes were incubated with secondary antibodies coupled to horseradish peroxidase for 1 h at room temperature. The membranes were washed three times with PBS-T, and immunoreactivity was detected using ECL reagents (Bio-Rad) and a Fusion SL3 instrument (Vilber Lourmat, Torcy, France). β-Actin (1:1000 dilution; Sigma-Aldrich) was used as a loading control. Plasma blots were stained with 0.1% Ponceau S for loading controls.

### Quantitative PCR

Total mRNA was extracted using TRIzol reagent (Life Technologies, Grand Island, NY, USA), according to the manufacturer’s instructions. Quantitative PCR was performed using a Bio-Rad CFX96. The PCR primers were as follows: ABCA1 FW, 5′-AAAACCGCAGACATCCTTCAG-3′ and ABCA1 RV, 5′-CATACCGAAACTCGTTCACCC-3′; SR-B1 FW, 5′-CGAAGTGGTCAACCCAAACGA-3′ and SR-B1 RV, 5′-CCATGCGACTTGTCAGGCT-3′. mRNA levels were normalized to those of GAPDH.

## Additional Information

**How to cite this article**: Nam, M. *et al.* Lipidomic Profiling of Liver Tissue from Obesity-Prone and Obesity-Resistant Mice Fed a High Fat Diet. *Sci. Rep.* **5**, 16984; doi: 10.1038/srep16984 (2015).

## Supplementary Material

Supplementary Data

## Figures and Tables

**Table 1:** Biochemical Characteristics of the ND, HFD-OR, and HFD-OP Mice.

	ND (n = 10)	HFD-OR (n = 8)	HFD-OP (n = 8)	*p*-Value (HFD-OR vs. HFD-OP)
Plasma parameters				
Total cholesterol (mg/dL)	130.28 ± 14.3	162.20 ± 6.4**	187.49 ± 16.5^††^	0.005
HDL cholesterol (mg/dL)	76.02 ± 7.2	86.32 ± 8.5*	90.01 ± 9.4^††^	0.442
HTR^a^	58.59 ± 4.57	53.24 ± 5.08*	48.25 ± 5.66^††^	0.085
non HDL-C (mg/dL)	54.26 ± 10.99	75.89 ± 9.2**	97.48 ± 16.8^††^	0.015
Apo-A1	32.56 ± 2.23	30.58 ± 2.40	30.86 ± 1.98	0.878
Glucose (mg/dL)	92.70 ± 10.2	113.38 ± 8.5*	139.38 ± 21.5^††^	0.007
Insulin (ng/mL)	1.28 ± 1.3	1.65 ± 0.4**	2.49 ± 0.9^††^	0.021
Glucagon (pg/mL)	163.55 ± 82.2	140.79 ± 82.1	197.71 ± 78.6	0.161
Leptin (ng/mL)	2.13 ± 1.7	24.90 ± 11.2**	66.05 ± 17.6^††^	0.001
Triglycerides (mg/dL)	88.72 ± 18.4	109.35 ± 23.4*	85.42 ± 19.3	0.050
HOMA-IR	4.59 ± 0.77	10.93 ± 1.95***	18.31 ± 6.50^†††^	0.037
Liver lipid content				
Hepatic triglycerides (mg/g liver)	31.35 ± 10.3	43.61 ± 6.5*	56.37 ± 12.3^††^	0.015
Hepatic cholesterol (mg/g liver)	1.72 ± 0.2	2.44 ± 0.5**	5.80 ± 2.1^††^	<0.001

Data are presented as the mean ± SD. *p*-values were calculated from Mann–Whitney *U*-tests. HFD-OR mice comparing to ND mice: *p < 0.05, **p < 0.01, **p < 0.001; HFD-OP mice comparing to ND mice: ^††^*p* < 0.01, ^†††^*p* < 0.001.

^a^HTR: (HDL cholesterol)/(total cholesterol) × 100 (%).
