# Supplementary material for: Lipidomic Profiling of Liver Tissue from Obesity-Prone and Obesity-Resistant Mice Fed a High Fat Diet
Source: Sci Rep. 2015 Nov 23;5:16984. doi: 10.1038/srep16984 (PMC4655311; doi:10.1038/srep16984)
Supplement: Supplementary Data [file srep16984-s1.doc]

**Lipidomic Profiling of Liver Tissue from Obesity-Prone and Obesity-Resistant Mice Fed a High Fat Diet**

Miso Nam^1,2^*, Myung-Sook Choi^3^*, Sunhee Jung^1,2^*, Youngae Jung^1^, Ji-Young Choi**^3^**, Do Hyun Ryu^2^, Geum-Sook Hwang^1^,**^4^**

^1^Integrated Metabolomics Research Group, Western Seoul Center, Korea Basic Science Institute, Seoul 120-140, Republic of Korea

^2^Department of Chemistry, Sungkyunkwan University, Suwon 440-746, Republic of Korea

**^3^**Department of Food Science and Nutrition, Kyungpook National University, Daegu, Republic of Korea

**^4^** Department of Life Science, Ewha Womans University, Seoul 120-750, Republic of Korea

**Corresponding author**

Do Hyun Ryu: Sungkyunkwan University, Suwon 440-746, Republic of Korea. Phone: +82 31 290 5931. Fax: +82 31 290 5967. E-mail: dhryu@skku.edu.

Geum-Sook Hwang: Korea Basic Science Institute, Seoul 120-140, Republic of Korea. Phone: +82-2-6908-6200. Fax: +82-2-6908-6239. E-mail: gshwang@kbsi.re.kr.

*These authors contributed equally to this work.

**Supplementary Table 1. Composition of the Mouse Diets**

| **Ingredient (g)** | **ND** | **HFD** |
| --- | --- | --- |
| Casein | 200 | 265 |
| Cornstarch | 397.486 | 0 |
| Sucrose | 100 | 90 |
| Maltodextrin | 132 | 160 |
| Cellulose | 50 | 65.6 |
| Soybean Oil | 70 | 30 |
| Lard | 0 | 310 |
| Mineral Mix*^a^* | 35 | 48 |
| Vitamin Mix*^b^* | 10 | 21 |
| Calcium Phosphate, Dibasic | 0 | 3.4 |
| TBHQ, Antioxidant | 0.014 | 0 |
| l-Cystine | 3 | 4 |
| Choline Bitartrate | 2.5 | 3 |
| Total (g) | 1000 | 1000 |
| Total energy (kcal) | 3800 | 5100 |

ND, normal diet (AIN-93G, 17% kcal fat); HFD, high-fat diet (60% kcal fat).

*^a^* AIN-93G: Mineral Mixture, *^b^*AIN-93G: Vitamin Mixture.

**
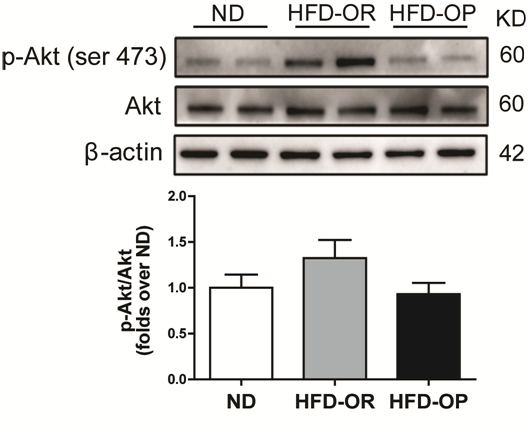
**

**Supplementary Figure 1. Expression of p-Akt and Akt in ND and HFD mice.** Representative western blot analysis and quantification of expression of p-Akt and Akt (ND, n = 5; HFD-OP, n = 5; HFD-OR, n = 5). β-Actin was used as a loading control.

**Supplementary** Table 2. Identified Lipid Metabolites with Significantly Different Concentrations between HFD-OP and HFD-OR Mice

| *m/z* | Ret. Time | Lipids | Classification | Ion species | VIP*^a^* | FC*^b^* | *p*-Value*^c^* |
| --- | --- | --- | --- | --- | --- | --- | --- |
| 594.5769 | 11.26 | Cer(d38:1) | Ceramide | [M+H]+ | 1.812 | 0.69 | 0.011 |
| 622.6089 | 11.69 | Cer(d40:1) |  | [M+H]+ | 1.554 | 0.85 | 0.011 |
| 620.5971 | 11.17 | Cer(d40:2) |  | [M+H]+ | 1.728 | 0.68 | 0.007 |
| 634.5397 | 9.58 | DG(36:4) | Diglyceride | [M+NH4]+ | 1.587 | 1.22 | 0.008 |
| 662.5717 | 10.79 | DG(38:4) |  | [M+NH4]+ | 1.475 | 1.73 | 0.015 |
| 468.3075 | 1.93 | LysoPC(14:0) | Lysophosphatidylcholine | [M+H]+ | 1.528 | 1.32 | 0.019 |
| 482.3238 | 2.3 | LysoPC(15:0) |  | [M+H]+ | 1.625 | 1.27 | 0.017 |
| 520.3397 | 2.12 | LysoPC(18:2) |  | [M+H]+ | 1.480 | 1.31 | 0.033 |
| 580.4356 | 5.73 | LysoPC(22:0) |  | [M+H]+ | 1.316 | 1.22 | 0.034 |
| 608.4634 | 6.68 | LysoPC(24:0) |  | [M+H]+ | 1.510 | 1.20 | 0.012 |
| 478.2913 | 2.21 | LysoPE(18:2) | Lysophosphatidylethanolamine | [M+H]+ | 1.606 | 1.47 | 0.007 |
| 702.5029 | 6.26 | PC(30:2) | Phosphatidylcholine | [M+H]+ | 1.956 | 1.30 | 0.001 |
| 720.5534 | 8.27 | PC(31:0) |  | [M+H]+ | 1.293 | 1.11 | 0.037 |
| 730.537 | 7.14 | PC(32:2) |  | [M+H]+ | 1.742 | 1.22 | 0.001 |
| 728.5221 | 6.47 | PC(32:3) |  | [M+H]+ | 2.042 | 1.30 | < 0.001 |
| 726.5022 | 6.15 | PC(32:4) |  | [M+H]+ | 1.629 | 1.36 | 0.007 |
| 756.5518 | 7.23 | PC(34:3) |  | [M+H]+ | 1.731 | 1.20 | 0.001 |
| 754.5362 | 7.01 | PC(34:4)^a^ |  | [M+H]+ | 1.591 | 1.20 | 0.007 |
| 754.5369 | 6.77 | PC(34:4)^b^ |  | [M+H]+ | 2.106 | 1.61 | < 0.001 |
| 784.5865 | 8.34 | PC(36:3) |  | [M+H]+ | 1.714 | 0.80 | 0.004 |
| 778.5329 | 6.82 | PC(36:6) |  | [M+H]+ | 1.406 | 1.17 | 0.035 |
| 816.6465 | 11.1 | PC(38:1) |  | [M+H]+ | 1.284 | 0.88 | 0.039 |
| 812.6135 | 9.39 | PC(38:3) |  | [M+H]+ | 1.960 | 0.69 | < 0.001 |
| 810.5972 | 8.39 | PC(38:4)^a^ |  | [M+H]+ | 1.258 | 0.76 | 0.015 |
| 810.601 | 8.96 | PC(38:4)^b^ |  | [M+H]+ | 1.492 | 0.91 | 0.010 |
| 808.583 | 8.01 | PC(38:5) |  | [M+H]+ | 1.388 | 0.86 | 0.021 |
| 806.0847 | 8.02 | PC(38:6)^a^ |  | [M+H]+ | 1.326 | 0.88 | 0.031 |
| 806.5658 | 8.4 | PC(38:6)^b^ |  | [M+H]+ | 1.389 | 0.87 | 0.021 |
| 804.5517 | 6.71 | PC(38:7) |  | [M+H]+ | 1.715 | 1.30 | 0.003 |
| 834.5985 | 9.4 | PC(40:6) |  | [M+H]+ | 1.782 | 0.76 | 0.001 |
| 850.5343 | 6.62 | PC(40:9) |  | [M+Na]+ | 1.464 | 1.15 | 0.035 |
| 717.5247 716.5211 | 8.28 8.29 | PE(34:2) | Phosphatidylethanolamine | [M+H]+ | 1.599 | 1.43 | 0.006 |
| 714.5043 | 7.4 | PE(34:3) |  | [M+H]+ | 1.701 | 1.40 | 0.003 |
| 738.5048 | 7 | PE(36:5) |  | [M+H]+ | 1.992 | 1.53 | < 0.001 |
| 736.4898 | 6.99 | PE(36:6) |  | [M+H]+ | 2.024 | 1.32 | < 0.001 |
| 774.5999 | 11.4 | PE(38:1) |  | [M+H]+ | 1.622 | 0.73 | 0.005 |
| 788.5067 | 4.52 | PE(39:1) |  | [M+H]+ | 1.386 | 1.35 | 0.045 |
| 794.5749 | 9.51 | PE(40:5) |  | [M+H]+ | 1.966 | 1.22 | < 0.001 |
| 906.6 | 8.03 | PI(38:3) | Phosphatidylinositol | [M+NH4]+ | 1.770 | 0.59 | 0.001 |
| 808.5129 | 6.68 | PS(38:6) | Phosphatidylserine | [M+H]+ | 1.735 | 1.25 | 0.005 |
| 701.5598 | 6.98 | SM(d34:2) | Sphingomyelin | [M+H]+ | 1.518 | 1.17 | 0.019 |
| 822.7525 | 23.12 | TG(48:1) | Triglyceride | [M+NH4]+ | 1.844 | 0.79 | 0.001 |
| 820.7368 | 21.95 | TG(48:2) |  | [M+NH4]+ | 1.414 | 0.84 | 0.025 |
| 836.7677 | 23.47 | TG(49:1) |  | [M+NH4]+ | 1.654 | 0.83 | 0.004 |
| 850.7888 | 23.77 | TG(50:1) |  | [M+NH4]+ | 1.408 | 0.81 | 0.026 |
| 853.7247 | 23.13 | TG(50:2) |  | [M+Na]+ | 1.926 | 0.75 | < 0.001 |
| 846.7548 | 22.06 | TG(50:3) |  | [M+NH4]+ | 1.497 | 0.80 | 0.016 |
| 862.7894 | 23.49 | TG(51:2) |  | [M+NH4]+ | 1.542 | 0.83 | 0.008 |
| 878.8084 | 23.77 | TG(52:1) |  | [M+NH4]+ | 1.603 | 0.82 | 0.006 |
| 876.7986 | 23.78 | TG(52:2) |  | [M+NH4]+ | 1.610 | 0.82 | 0.005 |
| 904.8322 | 24.21 | TG(54:2) |  | [M+NH4]+ | 1.639 | 0.73 | 0.003 |
| 896.7684 | 20.68 | TG(54:6)^a^ |  | [M+NH4]+ | 1.643 | 1.87 | 0.006 |
| 901.7253 | 20.67 | TG(54:6)^b^ |  | [M+Na]+ | 1.617 | 1.83 | 0.007 |
| 930.8474 | 24.19 | TG(56:3) |  | [M+NH4]+ | 1.405 | 0.69 | 0.025 |
| 926.8179 | 23.69 | TG(56:5) |  | [M+NH4]+ | 1.278 | 1.37 | 0.045 |
| 922.7827 | 21.32 | TG(56:7)^a^ |  | [M+NH4]+ | 1.411 | 1.54 | 0.031 |
| 922.7901 | 22.07 | TG(56:7)^b^ |  | [M+NH4]+ | 1.478 | 1.45 | 0.014 |
| 918.7539 | 17.59 | TG(56:9) |  | [M+NH4]+ | 1.552 | 2.09 | 0.014 |
| 946.7863 | 21.32 | TG(58:9) |  | [M+NH4]+ | 1.411 | 1.48 | 0.020 |

*^a^* Variable importance in the projection (VIP) was obtained by partial least-squares discriminant analyses (PLS-DAs) with a threshold of 1.0. *^b^* indicates fold change, and the value under 1 corresponds to the decreased relative concentration in HFD-OP vs. HFD-OR mice. *^c^ p*-Values were calculated from Mann–Whitney *U* tests with significance at *p* < 0.05.

**
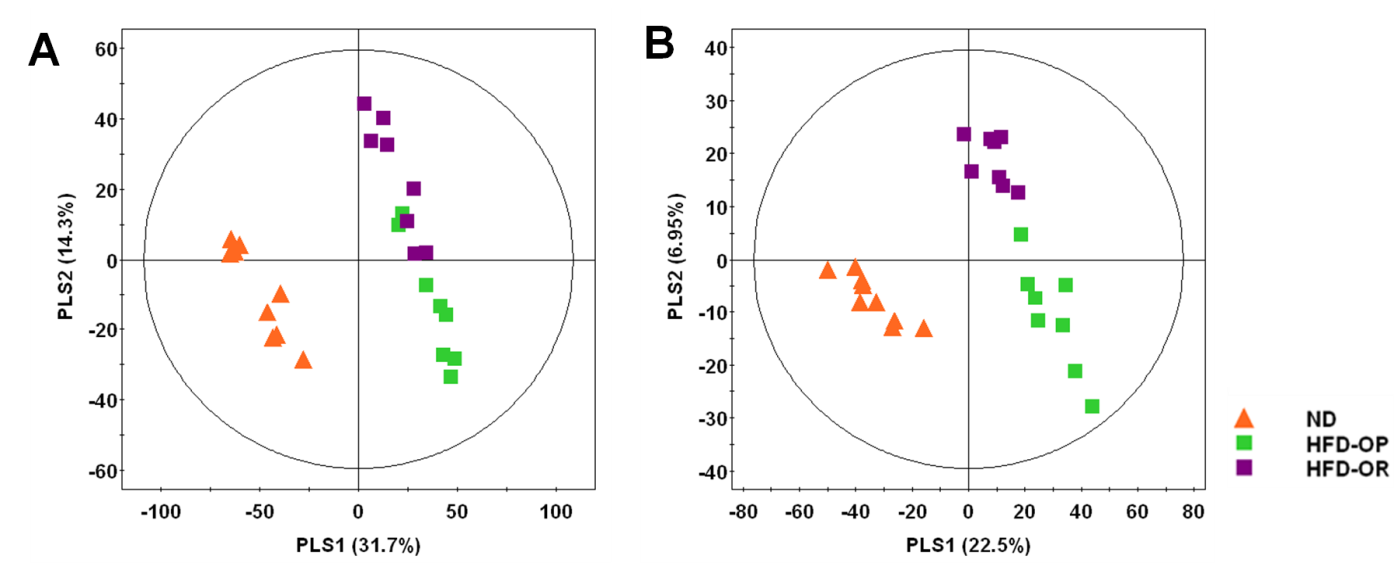
**

**Supplementary Figure 2.** Partial least-squares discriminant analysis (PLS-DA) score plots obtained from the UPLC/QTOF MS spectra of liver lipid extracts for global analysis. The different groups are clearly differentiated. (a) Positive mode (*R*^2^*X* = 0.7, *R*^2^*Y* = 0.987, *Q*^2^ = 0.889); (b) negative mode (*R*^2^*X* = 0.536, *R*^2^*Y* = 0.984, *Q*^2^ = 0.737).

**
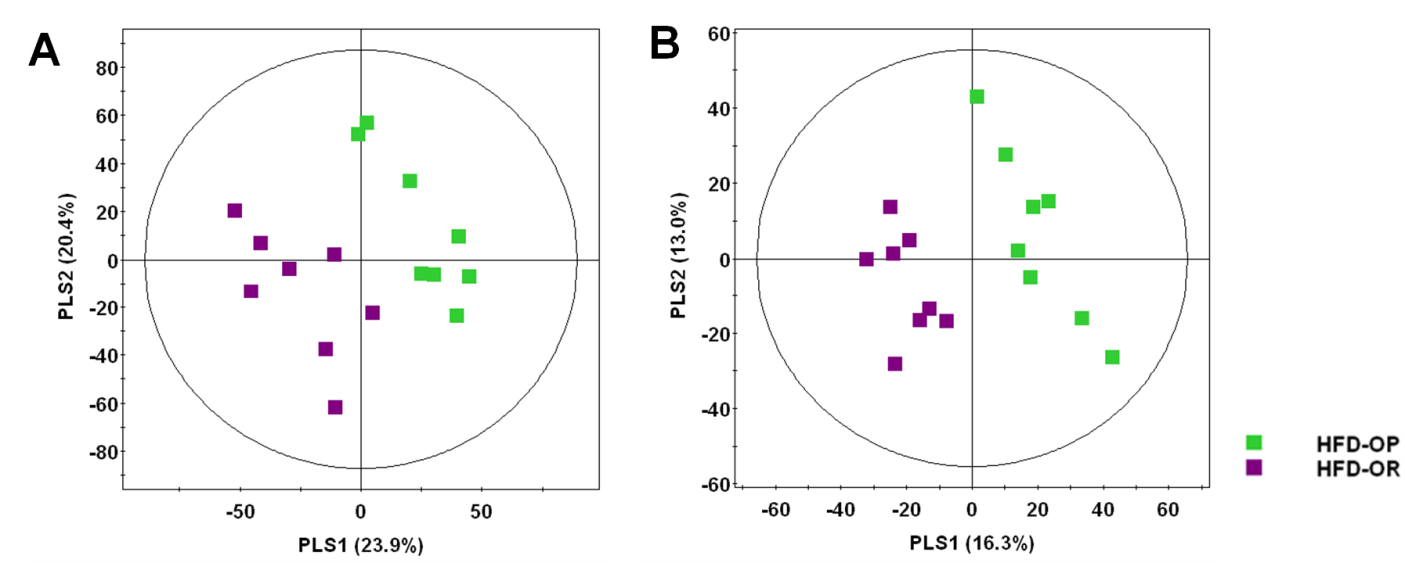
Supplementary Figure 3.** Partial least-squares discriminant analysis (PLS-DA) score plots obtained from the UPLC/QTOF MS spectra of liver lipid extracts for global analysis. HFD-OP and HFD-OR mice are clearly differentiated. (a) Positive mode (*R*^2^*X* = 0.644, *R*^2^*Y* = 0.99, *Q*^2^ = 0.868); (b) negative mode (*R*^2^*X* = 0.381, *R*^2^*Y* = 0.982, *Q*^2^ = 0.658).

**
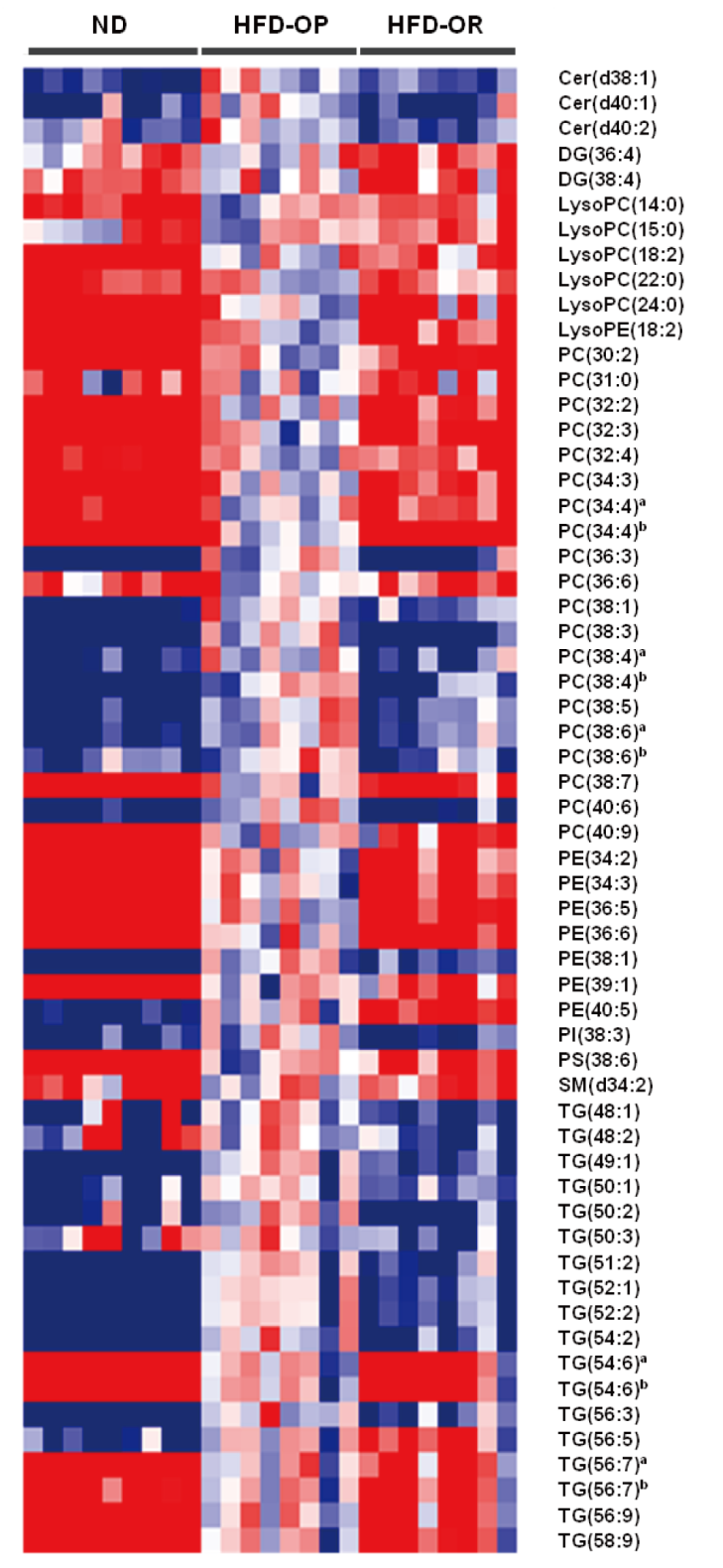
**

**Supplementary Figure 4.** A heat map of lipid profiling obtained from the UPLC/QTOF MS spectra of liver lipid extracts shows significant differences between the HFD-OP and HFD-OR mice. Each value in the map is a colored representation of a calculated *Z*-score. *Z*-scores were calculated based on the average of an HFD-OP group and its standard deviation as *Z*-score = [(MS intensity of lipid molecules – average of MS intensity of lipid molecules in HFD-OP group)/standard deviation of MS intensity of lipid molecules in HFD-OP group)]. Lipids are presented by the total number of carbon atoms and the total number of double bonds. Lipids with dissimilar MS/MS spectra but with identical numbers of carbon atoms and an equal number of double bonds are marked by ^a^ and ^b^.


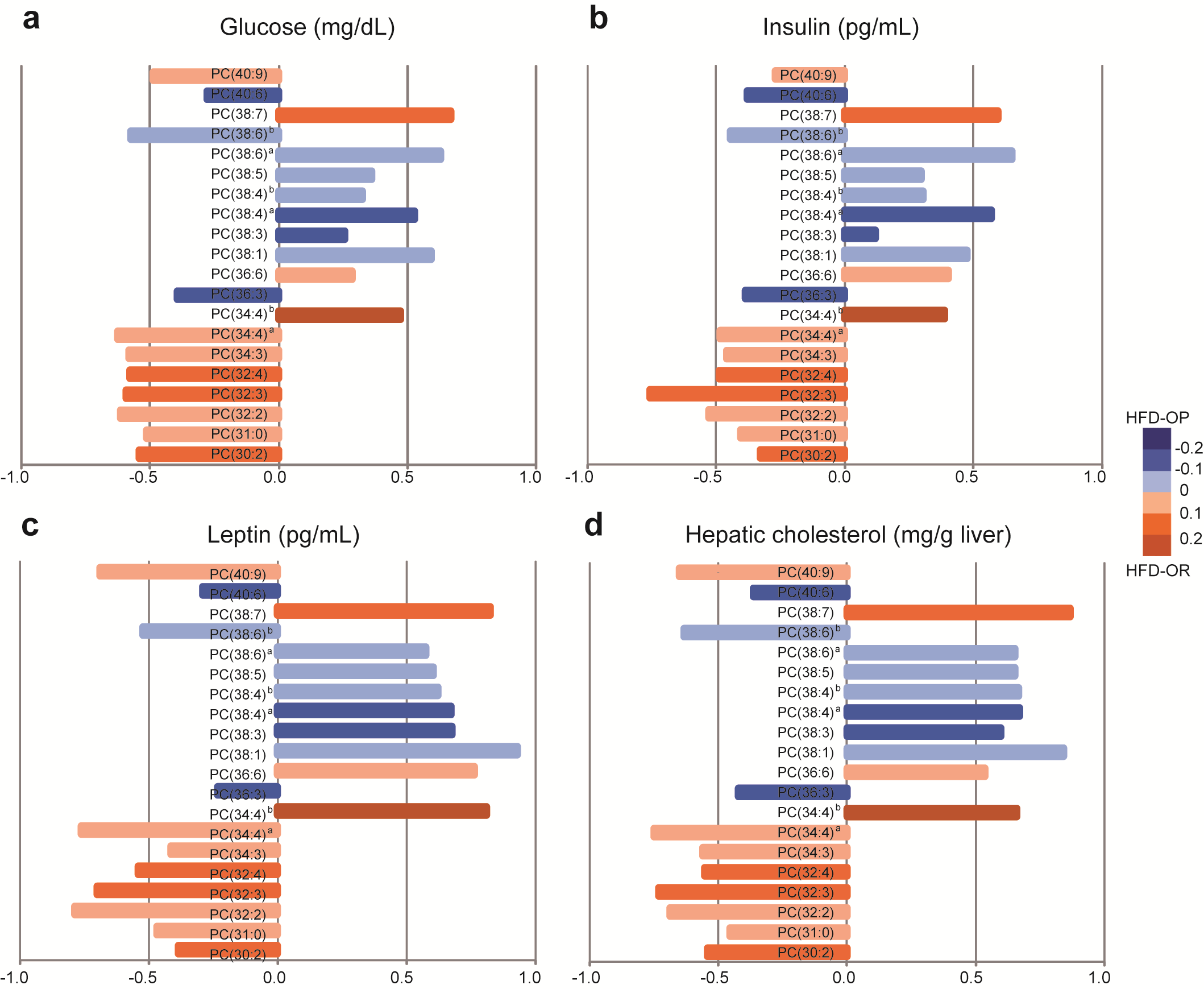


**Supplementary Figure 5.** Spearman’s rank correlations are shown for PC species with different biochemical characteristics: (a) glucose, (b) insulin, (c) leptin, and (d) hepatic cholesterol. Changes are displayed by color. A gradient of dark blue in proportion to each lipid species showing a high concentration in HFD-OP mice and a gradient of dark orange in proportion to each lipid species showing a high concentration in HFD-OR mice. PC species are presented by the total number of carbon atoms and the total number of double bonds. Lipids with dissimilar MS/MS spectra but with identical numbers of carbon atoms and an equal number of double bonds are marked by ^a^ and ^b^.
